# Case report: Hepatectomy with Rex bypass for a child with hepatoblastoma and portal vein thrombosis

**DOI:** 10.3389/fped.2023.1203212

**Published:** 2023-05-18

**Authors:** Norihiko Kitagawa, Masato Shinkai, Fumio Asano, Yukihiro Tsuzuki, Kyoko Mochizuki, Hidehito Usui, Yuma Yagi, Akio Kawami, Kazuyoshi Okumura, Tomoko Yokosuka, Hiroaki Goto, Kumiko Nozawa, Mio Tanaka

**Affiliations:** ^1^ Department of Surgery, Kanagawa Children’s Medical Center, Yokohama, Japan; ^2^ Department of Hematology/Oncology, Kanagawa Children’s Medical Center, Yokohama, Japan; ^3^ Department of Radiology, Kanagawa Children’s Medical Center, Yokohama, Japan; ^4^ Department of Pathology, Kanagawa Children’s Medical Center, Yokohama, Japan

**Keywords:** hepatoblastoma (HBL), Rex bypass, portal occlusion, hepatectomy, liver preservation

## Abstract

Pediatric liver tumors with portal vein obstruction are often candidates for liver transplantation. However, lifelong use of immunosuppressants and invasiveness to healthy donors in the case of living-donor liver transplantation is inevitable. Moreover, when lung metastasis is involved, the lung recurrence rate after liver transplantation is still high. Therefore, transplantation should be avoided as much as possible. In cases of tumors in the right lobe of the liver, complete resection of the portal vein trunk may be possible by creating a Rex bypass, but with the original method, end-to-side anastomosis to the umbilical portal vein is difficult in small children. We report a case of a 2-year-old girl with hepatoblastoma in whom a Rex shunt was created by end-to-end anastomosing the recanalized umbilical vein to the portal vein stump with interposing a vein graft, and the right lobe was successfully resected along with the tumor.

## 1. Introduction

Pediatric liver tumors may be associated with portal vein obstruction, in which case, liver transplantation in a timely fashion is often indicated. However, liver transplantation involves the lifelong administration of immunosuppressive drugs, and living-donor liver transplantation poses problems such as invasiveness to healthy donors. Therefore, maximal liver preservation is highly required. In the case of right-lobe hepatic tumors that occlude the portal vein trunk by a tumor thrombus, liver transplantation may be avoided if blood flow to the residual left lobe can be secured using a mesenterico/porto-Rex bypass. To address the difficulty of bypass anastomosis in small children, we have adopted a method of recanalizing the umbilical vein in the round ligament of the liver and anastomosing it to the superior mesenteric vein or portal vein trunk. Here, we report our experience of using this method to perform hepatectomy in a child with hepatoblastoma occluding the portal vein.

## 2. Case description

**Figure 1:**
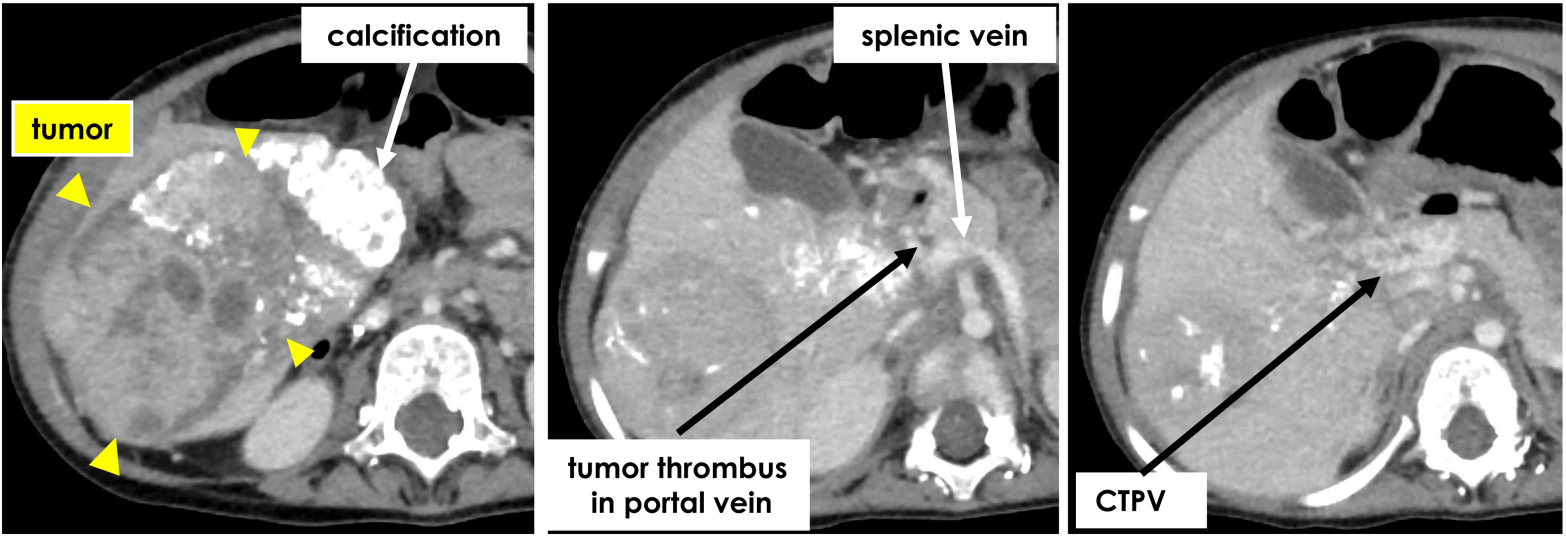
Contrast-enhanced computed tomography after chemotherapy showed tumor thrombus in portal vein trunk and growth of cavernous transformation.

**Figure 2:**
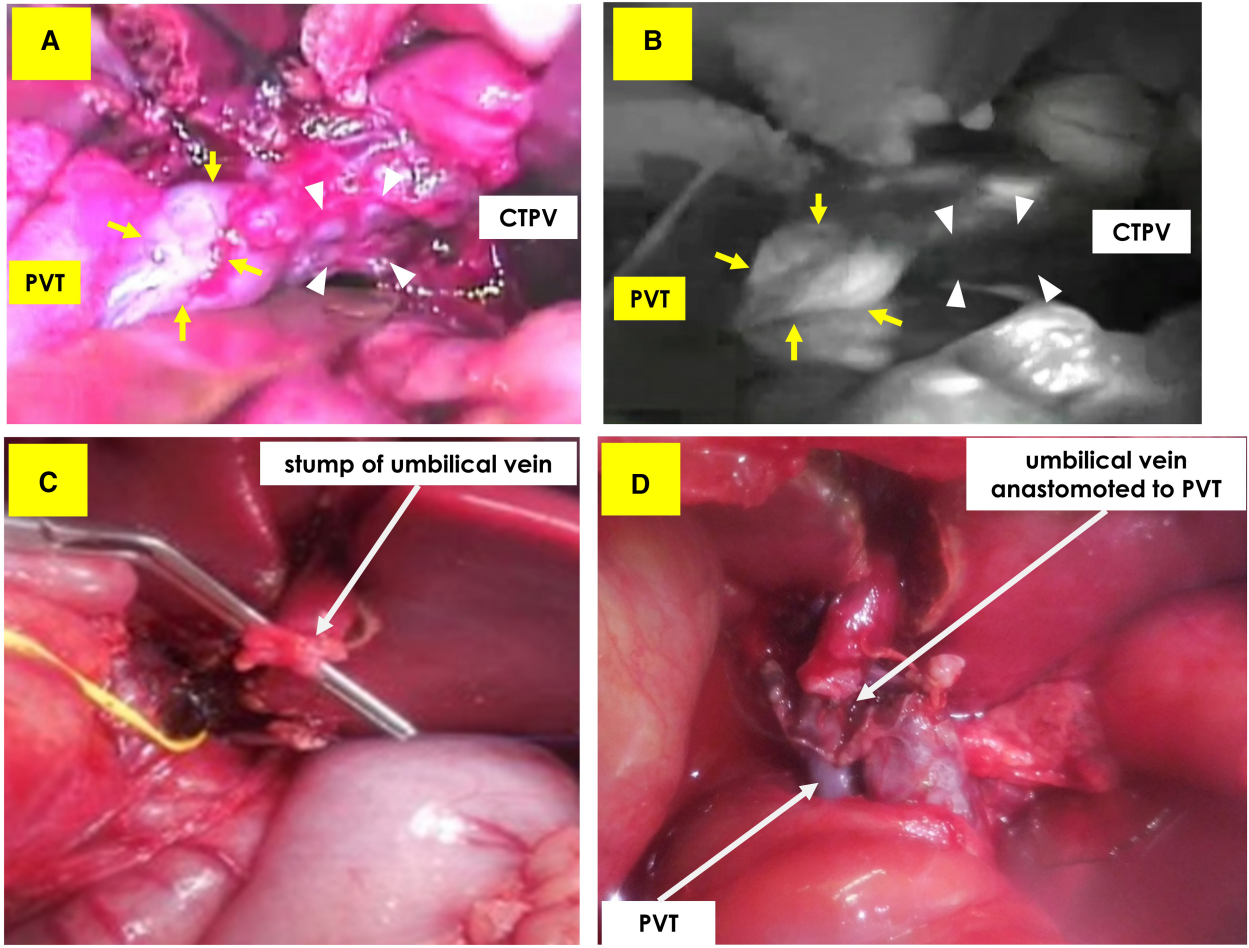
Upper: intraoperative ICG fluorescence method. (**A**) Visible light photo shows portal vein trunk (PVT: arrow) and cavernous transformation of the portal vein (CTPV: triangle). (**B**) Positive fluorescence was detected only from PVT, suggesting existence of viable tumor tissue. No fluorescence was detected from CTPV. Lower: Making a Rex bypass. (**C**) The umbilical vein was detected in the round ligament. (**D**) Recanalized umbilical vein was anastomosed to portal vein trunk stump.

**Figure 3:**
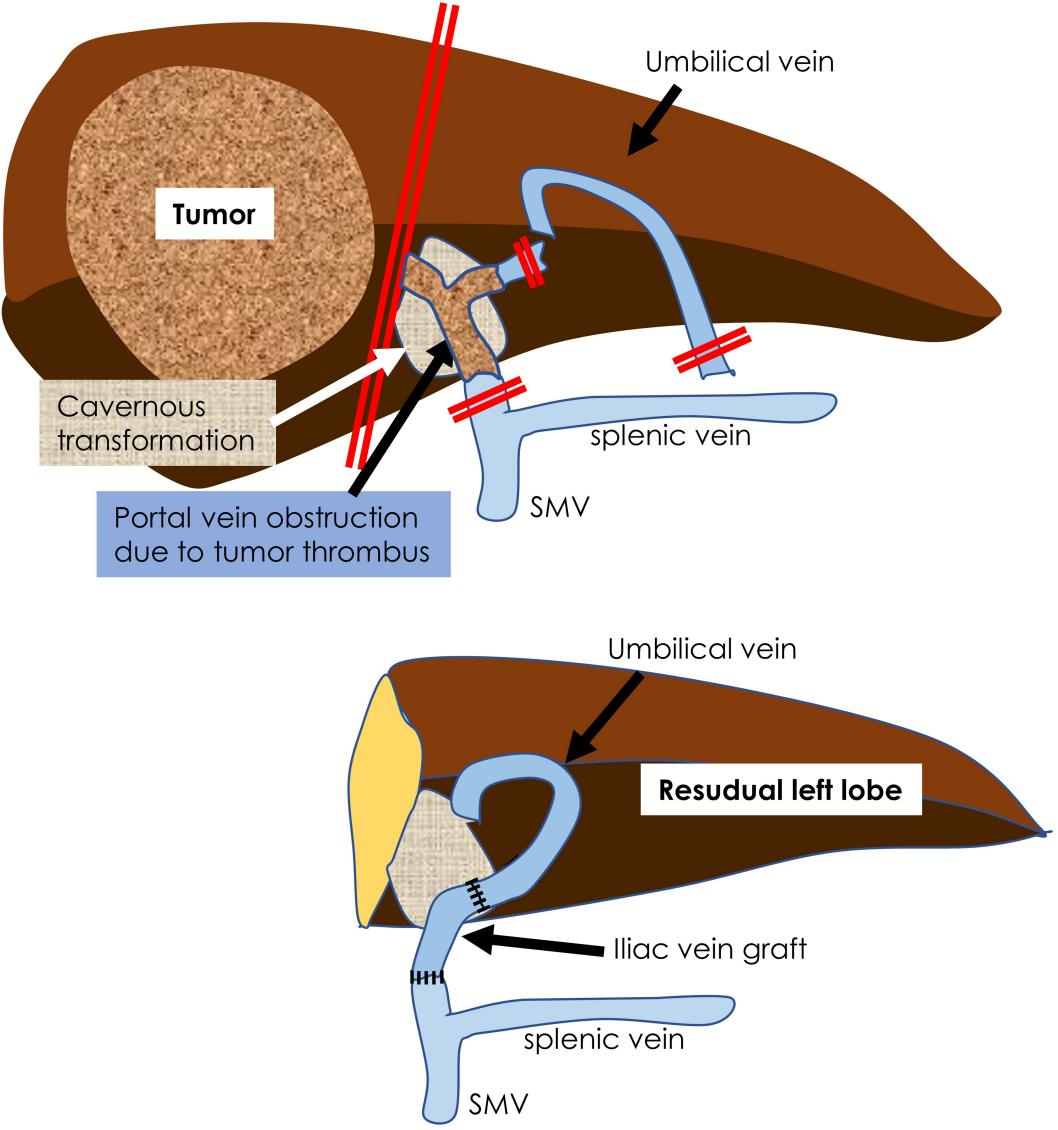
A schema of this operation is shown. The main trunk of the portal vein containing the tumor thrombus was resected. The umbilical vein leading to the portal umbilical portion was recanalized and an iliac vein graft was interposed and anastomosed to the portal vein stump.

A little girl of two years and two months (weight: 6,532 g) was referred to our hospital after her mother noticed a right upper abdominal distension; and a liver tumor was diagnosed at a neighborhood pediatric clinic. She was a triplet weighing 342 g at birth and had chronic lung disease, hypothyroidism, and right retinal detachment. A 9.5 cm × 7 cm × 10 cm tumor was found in the right lobe of her liver on contrast-enhanced computed tomography (CT), the main portal vein and bilateral first branches were obstructed by a tumor thrombus, and a cavernous transformation had developed. No tumors were present in the left or caudate lobes. Her initial serum alpha-fetoprotein level was 3,220,400 ng/ml; and her serum ALT and AST levels were 86 and 59, respectively. The patient was diagnosed with hepatoblastoma (mixed epithelial and mesenchymal type, PRETEXT II) on biopsy. CT showed a nodule with a diameter of 2 mm in the right lower lung lobe, suggestive of lung metastasis. Chemotherapy was initiated with the SIOPEL-4 regimen, which resulted in tumor shrinkage, but no improvement in the portal obstruction (Figure 1). The lung metastasis disappeared after chemotherapy. Three months after diagnosis, she underwent laparotomy. Intraoperatively, the indocyanine green (ICG) fluorescence demonstrated fluorescence in the main trunk of the portal vein, suggesting the presence of viable tumor tissue (Figure 2). In contrast, no fluorescence was observed in her cavernous transformation of the portal vein (CTPV). We decided to extirpate the portal vein trunk and preserve the CTPV to avoid damage to the bile duct and preserve hepatopetal collaterals. The right hepatic artery, right hepatic vein, and right hepatic duct were ligated and divided, followed by resection of the right lobe of the liver and the portal vein trunk. The middle hepatic vein, the common bile duct, and CTPV were preserved. The left branch of the portal vein was ligated and divided near the umbilical portion and at the trunk. However, because hepatopetal blood flow through the CTPV was insufficient for the remaining left lobe, a Rex bypass was constructed by anastomosing the umbilical vein directly to the portal trunk stump (Figure 2). The quality of the umbilical vein, as a bypass vessel, was evaluated by means of the strength of the backflow after dilation. Furthermore, after anastomosis, the blood flow velocity was assessed using Doppler ultrasonography. After transfer to the ICU, the portal vein circulation became poor; therefore, laparotomy was performed again. An external iliac vein graft was interposed and re-anastomosed, resulting in a good blood supply (Figure 3). Danaparoid was administered for four days after surgery. It was substituted by dipyridamole, which was discontinued due to eosinophilia one month after the procedure. As the blood flow in the bypass vessel was good, it was not resumed. Chemotherapy was, however, administered for four months after the termination of dipyridamole use. One year later, she was diagnosed with extrahepatic sclerosing cholangiopathy and underwent a choledochojejunostomy. There has been no tumor recurrence for up to 5 years after the operation.

## 3. Discussion

The frequency of hepatoblastoma associated with portal vein invasion is approximately 10% (1). In most cases, liver transplantation is indicated if the portal vein trunk invasion is not resolved by chemotherapy (2). In the present case, pulmonary metastases were present at the initial presentation. Since the recurrence rate of lung metastasis after liver transplantation is still high (3), we wanted to avoid liver transplantation, if possible.

When the portal trunk is occluded by a tumor thrombus, hepatopetal blood flow is preserved by CTPV. In this case, resection of the main portal vein and CTPV after the preceding creation of the Rex shunt may be considered as an alternative, but resection of the CTPV will result in difficulty controlling bleeding, and there is a risk of bile duct damage (4); therefore, we decided to resect only the main trunk of the portal vein. We used the ICG fluorescence method (5) to confirm the absence of tumor invasion on CTPV and determined that preservation was possible.

The mesenterico/porto-Rex shunt is considered the most physiological type of portal vein bypass surgery (6). It is usually used for diseases such as extrahepatic portal vein occlusion (7–9), Kumada et al. reported the use of this technique in adult patients with hepatic hilar tumors (10). However, end-to-side anastomosis with the umbilical portion of the left portal vein is technically difficult in small children. Therefore, we used end-to-end anastomosis of the umbilical vein (UV) with the portal vein stump (11). The UV in the round ligament of the liver that continues from the left portal vein is recanalized with serial dilators and anastomosed to the portal vein trunk or the superior mesenchymal vein. This method allows for end-to-end anastomosis and is technically feasible. Anastomosis is relatively easy, even if the graft vessels are interposed. Therefore, our method may be considered an alternative to the well-received standard method of Rex bypass creating end-to-side anastomosis with the umbilical portion of the left portal vein.

Notably, the incidence of thrombotic vascular occlusion increases with the use of long UV; therefore, postoperative anticoagulant and antiplatelet therapy may be used (6, 12). Therefore, postoperative monitoring by ultrasonography is important.

We used this method in a neonatal case of benign hepatic mesenchymal hamartoma, as previously reported (13). Briefly, an infant weighing 2.96 kg was found to have a giant mesenchymal hamartoma with abdominal compartment syndrome, and emergency right hepatic lobectomy was performed. Part of the portal vein was occluded by the tumor; therefore, a Rex shunt was constructed by anastomosing the umbilical vein to the stump of the portal vein. Ten years after the surgery, no recurrence was observed. In this patient, we performed this procedure for malignant hepatoblastoma and report the results.

**Figure 4:**
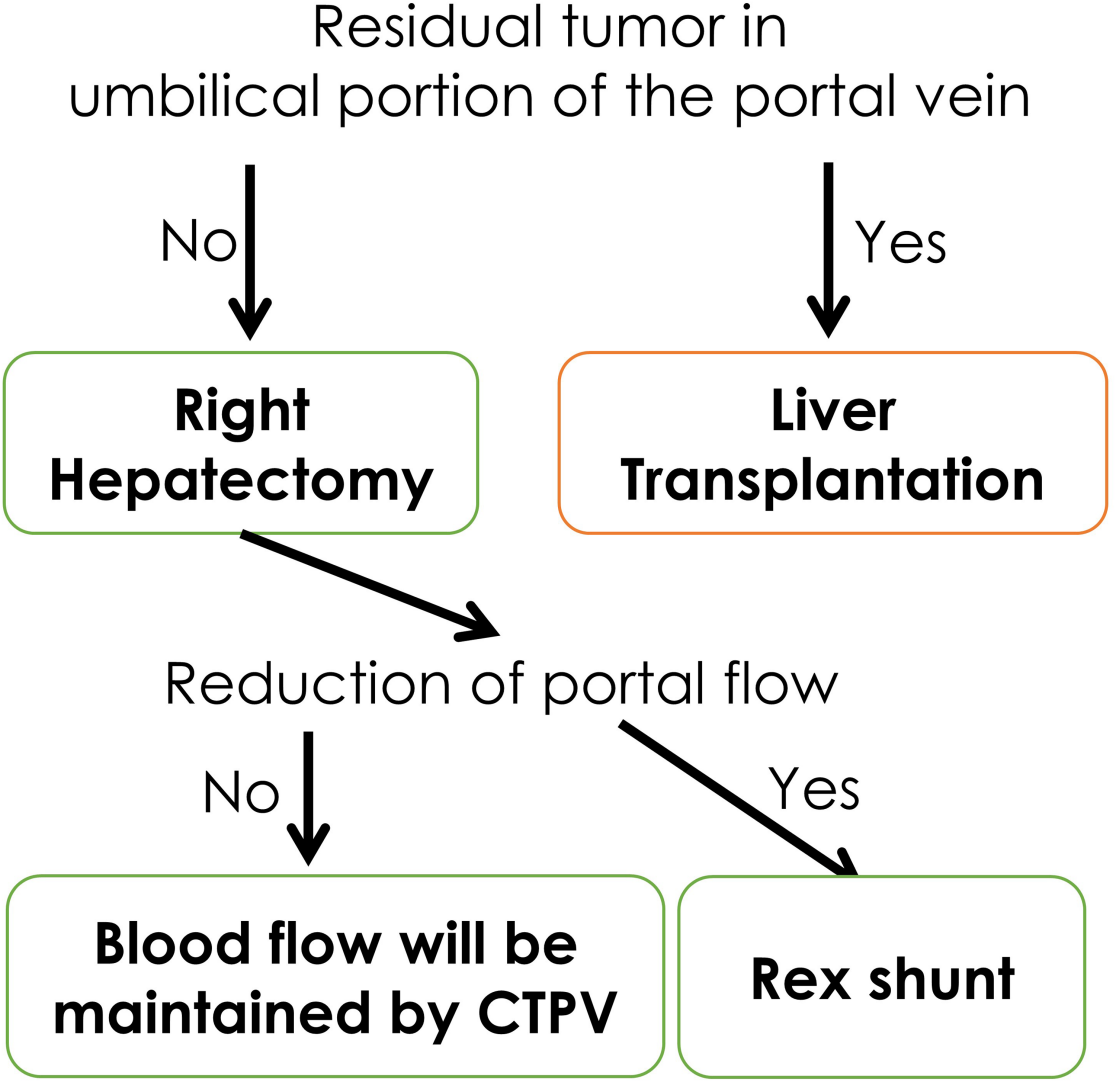
Our strategy for right lobe hepatoblastoma with portal vein thrombus.

Here, we present a strategy for treating malignant hepatic tumors originating in the right hepatic lobe with a primary portal vein tumor thrombus (Figure 4). In conclusion, a Rex shunt can be used to preserve the left hepatic lobe and avoid liver transplantation in cases of hepatoblastoma with a portal vein tumor thrombus. Further follow-up is required to assess the long-term efficacy and safety of this surgical procedure.

## Data Availability

The original contributions presented in the study are included in the article, further inquiries can be directed to the corresponding author.
